# Incidence of spine-related diagnoses in Danish children: a nationwide registry-based study of hospital data

**DOI:** 10.1007/s00431-025-06247-w

**Published:** 2025-06-21

**Authors:** Freja Gomez Overgaard, Mette Wod, Lise Hestbæk, Henrik Hein Lauridsen, Søren Francis Dyhrberg O’Neill, Michael Swain, Casper Nim

**Affiliations:** 1 Medical Spinal Research Unit, Spine Centre of Southern Denmark, University Hospital of Southern Denmark, Kolding, Denmark; 2 Department of Regional Health Research, University of Southern Denmark, Odense, Denmark; 3 Department of Chiropractic, Faculty of Medicine, Health and Human Science, Macquarie University, Sydney, Australia; 4 Center for Clinical Epidemiology, Odense University Hospital, Odense, Denmark; 5 Research Unit of Clinical Epidemiology, Department of Clinical Research, University of Southern Denmark, Odense, Denmark; 6 Center for Muscle and Joint Health, Department of Sports Science and Clinical Biomechanics, University of Southern Denmark, Odense, Denmark; 7 The Chiropractic Knowledge Hub, Odense, Denmark

**Keywords:** Pediatrics, Spinal diseases, Epidemiology, Diagnoses, Registries, Healthcare utilization

## Abstract

This study investigated the annual incidence rates of spine-related hospital diagnoses in Danish children aged 0–17 years from 2009 to 2021. It aimed to assess trends stratified by diagnostic groups, hospital departments, and residential regions. A nationwide register-based cohort study was conducted using data from the Danish National Patient Register and census data. Spine-related diagnoses were identified through International Classification of Disease (ICD-10) codes and categorized into five groups: critical, whiplash, radiating, structural, and regional. Incidence rates (IR) per 100,000 children were calculated and stratified by diagnostic group, hospital department, and region. Temporal trends were visualized using descriptive statistics. The study identified 43,073 children with 78,304 spine-related diagnoses. The median age was 13 years with interquartile range of 5.3, and 55% were female. IRs remained stable over the 13-year period. Whiplash and structural diagnoses decreased after 2015, while regional diagnoses increased until stabilizing in 2019. Regional disparities were noted, with the capital region having higher IRs than other regions. Most diagnoses were managed within orthopedic departments. *Conclusion*: This study highlights stable IRs of pediatric spine–related diagnoses despite significant changes in diagnostic practices, reflecting a potential shift toward a biopsychosocial approach in hospital care. Regional disparities and variability in diagnostic coding practices emphasize the need for standardized protocols and equitable care pathways. Further research could validate coding practices, investigate care trajectories, and explore long-term outcomes to optimize pediatric spine care.

**Table:** 

What is Known: • Pediatric spinal pain is increasingly reported in epidemiological surveys, with substantial health burden. • Hospital-based diagnostic patterns in children remain largely unexplored.
What is New: • This is the first national registry-based study of spine-related hospital diagnoses in Danish children. • Marked regional variation and changing departmental patterns suggest a need for standardization.

**Supplementary Information:**

The online version contains supplementary material available at 10.1007/s00431-025-06247-w.

## Introduction

### Background

Pediatric spinal pain (pain arising from the neck, mid-, and lower back) is a growing health concern in youth, with prevalence estimates ranging from 20 to 30% in school-aged populations over a 12-month period in Europe and North America [1–4]. In Denmark, a prospective population-based study found that approximately one-third of spinal pain episodes in children and adolescents (hereafter referred to as children) were of non-trivial severity, often requiring assessment by allied health practitioners [2, 5]. Additionally, gender differences have been observed, particularly between ages 11 and 14, where 10% of boys and 14% of girls report experiencing spinal pain [6, 7].

There is evidence that children with spinal pain have a considerable level of healthcare utilization. Children with spinal pain commonly seek care in primary healthcare settings [8]. In Denmark, 8% of 13-year-olds and 34% of 15-year-olds seek healthcare for musculoskeletal pain [9]. However, little research has been conducted on the hospital management of children with spinal pain, aside from conditions such as scoliosis, severe trauma, and other specific diagnoses requiring specialist assessment [10].

The hospital system in Denmark is made up primarily of government-run, publicly funded hospitals, with only a smaller part being private enterprises. Pediatric patients with spinal pain will be seen in a hospital setting only upon qualified referral, e.g., from general practitioners or consultant specialists, to a specialized department or as a walk-in to the emergency department. However, there is limited knowledge about how children and adolescents with spinal pain are managed within the hospital system. While it is likely that they are predominantly seen in orthopedic or pediatric hospital departments, there is a lack of data on where spine-related diagnoses are assigned and whether management strategies vary across regions.

Considering the high burden of spinal pain reported in children [6], the associated socioeconomic costs, and the limited knowledge regarding healthcare utilization in this population, pediatric spinal pain deserves more focus. Identifying the number of children assessed in hospital settings, the age groups affected, and regional disparities will provide a more comprehensive understanding of the utilization of secondary sector healthcare services by children with spinal pain.

### Objectives

To investigate the annual incidence rate of pediatric spine–related diagnoses within Danish Hospital Departments based on register-based diagnostic codes from 2009 to 2021. Moreover, to assess differences across the Danish regions and between different spine-related diagnostic groups.

## Method

### Study design and setting

This study design is a historical nationwide register-based cohort study. The study is reported as per the Strengthening the Reporting of Observational Studies in Epidemiology (STROBE) guidelines [11]. In accordance with Danish law and confirmed by the Ethics Committee of Southern Denmark [12], registered-based studies are exempt from ethical approval. The study was registered in the internal directory of The Region of Southern Denmark (ID: 23/33311). The data management and analyses were conducted on Statistic Denmark’s (DST) servers between October 2023 and December 2024, with the authorization of the Region of Southern Denmark’s research support unit OPEN [13].

The Danish National Patient Register only includes data from publicly funded hospitals. Diagnoses recorded in private hospitals were not captured in this study.

### Data sources/measurement

Data were retrieved from relevant Danish national registries:

- The Danish Civil Registration System (CRS): Launched in 1968, the CRS records all individuals born in Denmark or residing legally for 3 months or more, assigning each a unique identifier number (CPR) [14]. Key variables include date of birth, parents’ CPR numbers, and parents’ status (deceased, expatriated, annulled, etc.).
- The Danish National Patient Register (DNPR): Established in 1976, the DNPR records all clinical activities in somatic (i.e., non-psychiatric) hospital departments, including data on diagnoses, municipalities, admission times, medical specialty, and hospital departments [15]. The DNRP was used to identify the case population.
- Census data from Denmark Statistics was used to access data on the population at risk (see below) [16].

### Participants

The study population of interest included all individuals with at least one hospital clinical activity recorded in the DNPR with a spine-related diagnosis as either a primary (A) or secondary (B) diagnostic code given between 2009 and 2021, or with no spine-related diagnosis from 2007 to 2009 to reduce selection bias. The included spine-related diagnoses from the International Classification of Diseases (ICD-10) codes [17] were M4*: (e.g., M436 torticollis), M5*: (e.g., M542 cervical spinal pain), M96*: (e.g., M964 lordosis after surgery), M99*: (e.g., M995 discus stenosis), and S13*: (e.g., S134 distortion of cervical spine). In all cases, the first diagnosis (index diagnosis) was used for classification. Each individual could receive multiple spine-related diagnoses during a single hospital encounter or across multiple encounters. If multiple spinal diagnoses were recorded at the same initial time point, A codes were prioritized and ranked based on potential severity in the following order: “Critical,” “Whiplash,” “Radiating,” “Structural,” and “Regional” (see under “ Classification of the index diagnoses”).

The “population at risk” included all those under the age of 18 living in Denmark from 2009 to 2021, retrieved through the census database [16] and subtracted from the case population. The starting point of 2007 was chosen due to the 2007 Municipal Structural Reform in Denmark, which organized the national healthcare system into five Danish regions. The exclusion criteria were*:*

- Patients with replacement of the status for the Central Person Register (CPR) identifier at the index date (code: 20 (inactive), 30 (annulled), 50 (double), 60 (changed), and 70 (missing)).
- Patients without Danish residence at the index date (codes: 05 and 07 (Greenland)).
- Patients who had died or emigrated prior to the index date (code: 80 (emigrated) and 90 (dead)).

### Variables

- Demographic:
  o Sex, as ascribed at birth based on CPR.
  o Age at the index diagnosis date.
- Geographic/institutional:
  o Region: Regions were derived from the hospital code and classified as residential regions (Capital, Southern, Central, Northern, and Zealand).
  o Department: The hospital department where index diagnosis was recorded. These were classified into broader groups (e.g., pediatric, neurology). Ambiguous hospital departments were recorded as the “Other” category (e.g., rehabilitation).
- Time
  o The time point of the index diagnosis was categorized into annual quarters.

### Quantitative variables

#### Classification of the index diagnoses

The classification into five diagnostic groups was developed by authors FGO and CN based on clinical relevance, etiological similarity, and practical grouping of ICD-10 codes. While no formal reliability testing was conducted, the classification was finalized in consensus among the co-authors to ensure consistency. A complete list of codes is provided in Appendix 1 (list of categories and diagnoses). The selected categories reflect distinctions frequently emphasized in clinical guidelines and research, including structural deformities, traumatic injuries, radicular symptoms, and non-specific pain syndromes, pediatric emergency diagnosis grouping frameworks, and spine-related care models such as the Global Spine Care Initiative [18–20].

- Critical: conditions characterized by severe pathological changes requiring intensive medical attention or associated with significant structural, inflammatory, traumatic, or surgical complications (e.g., M46.4 spondylodiscitis, M80.4 vertebral osteonecrosis).
- Whiplash: conditions characterized by trauma diagnoses related to whiplash, all under the diagnosis code S13 (e.g., S13.4 whiplash syndrome).
- Radiating: conditions characterized by confirmed radiating pain in the extremity (e.g., M47.9 spondylosis, M50.1 cervical disc herniation).
- Structural: conditions involving structural spine deformities (e.g., M14.1 scoliosis).
- Regional: conditions specific to the spine, with or without listed traumatic or degenerative structures (e.g., M54.5 non-specific spinal pain).

In cases where a diagnosis could theoretically belong to more than one group, categorization was based on the most clinically dominant aspect of the code, as reflected in its ICD-10 description. If a child had multiple spine-related codes recorded at the index time point. Group assignment was prioritized using a predefined hierarchy based on clinical severity: critical > whiplash > radiating > structural > regional. This approach aimed to ensure internal consistency in group allocation.

In this study, we use the term “non-specific” to refer to diagnostic codes that describe spinal pain without a clearly identifiable structural, traumatic, or neurological cause. This differs from the ICD-10 label “unspecified,” which refers to lack of coding detail rather than clinical interpretation.

#### Classification of hospital departments

Hospital departments relevant to pediatric spine–related diagnoses were categorized into eight groups. Authors FGO and CN developed the classification of hospital department groups. The hospital groups were created to provide an overview of how diagnoses were distributed across hospital departments. Group definitions are defined below (Appendix 2, list of departments).

- Pediatric: includes generalized pediatric departments. While child psychiatry is generally not recorded in DNPR, it was included in this classification to account for possible cases recorded within general pediatric hospital settings..
- Orthopedic: includes all orthopedic departments.
- Emergency: includes all emergency departments, providing immediate care for acute and urgent illnesses and injuries.
- Rheumatology: includes all rheumatology departments.
- Neurology: includes neurology and neurophysiology departments.
- Neurosurgery: includes all neurosurgery departments.
- Other: includes departments that do not fall within the definition of the other groups.

### Statistical analysis

Descriptive statistics were reported for the case population on age, sex, region, and hospital department. Also reported was the total number of spine-related diagnoses per child. Index diagnoses were selected to estimate annual quarterly incidence rates (IR) of diagnoses (yes/no). Incidence rates were calculated for each annual quarter by dividing the number of new cases with the population at risk for each period per 100,000 children who did not have a spine-related diagnosis since 2007. Confidence intervals (95%CI) were calculated using normal approximation to provide a statistical measure of uncertainty around the IR estimates. The incidence rates were stratified based on (i) the administrative region where the diagnosis was established and (ii) the diagnostic groups and iii) the department group where the diagnosis was established.

The completeness of Danish national registries minimized the number of missing data, so any missing data were omitted from the analysis. This follows practices commonly used in registry-based incidence studies [14]. All statistical analyses were conducted in R (version 4.3) [21].

## Results

### Identification and demographics

A total of 43,073 unique children were identified with spine-related diagnoses between 2009 and 2021; seven children had missing registry data and were excluded from the analysis. The total number of spinal diagnoses provided across individuals was 78,304, as some individuals have multiple diagnoses recorded either within the same hospital encounter or across different encounters. The population’s median age was 13 years (interquartile range (IQR) = 5.3 years, range 0–17 years), and 55% were female (Table 1). The three most frequent diagnoses were distortion of the cervical spine (15,005; 35%), thoracic scoliosis (5123; 12%), and unspecified spinal pain (5081; 12%) (Table 2).

**Table 1:** Demographic characteristics of 43,073 Danish children identified with spine-related diagnoses

Characteristic	***n*** (%)/median (interquartile range (IQR))
Age	Median: 13.0 years (Q25–Q75: 9; 15)
Sex (female)	23,608 (55%)
Danish regions	
Capital	13,995 (32%)
Southern	10,524 (24%)
Central	8989 (21%)
Zealand	5895 (14.5%)
Northern	3670 (8.5%)
Hospital department	
Orthopedic	22,047 (51%)
Other	9890 (23%)
Pediatric	6231 (14%)
Neurosurgery	2213 (5.1%)
Rheumatology	1750 (4.1%)
Emergency	886 (2.1%)
Neurology	56 (0.1%)
Diagnostic groups	
Whiplash	15,397 (36%)
Structural	13,634 (32%)
Regional	12,790 (30%)
Critical	763 (1.8%)
Radiating	489 (1.1%)

**Table 2:** The five most frequent spine-related diagnoses based on total number of diagnoses (*N* = 78,304)

**Diagnostic code**	**Description**	**Frequency**	**Percentage of total diagnoses**
S134	Distortion of the cervical spine	15,005	34.8%
M411	Thoracic scoliosis	5123	11.9%
M549	Unspecified spinal pain	5081	11.8%
M419	Other forms of scoliosis	3446	8.0%
M436	Torticollis	3054	7.0%

### Incidence rates

**Fig. 1:**
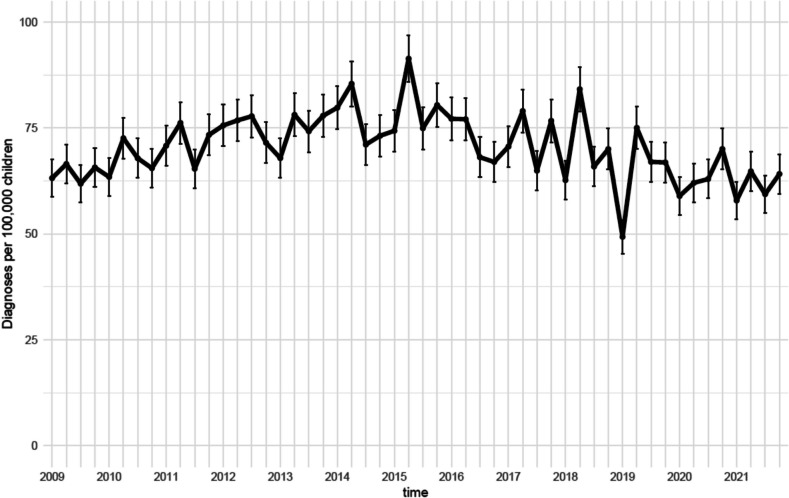
Annual quarterly incidence rates of spine-related diagnoses (2009–2021)

**Fig. 2:**
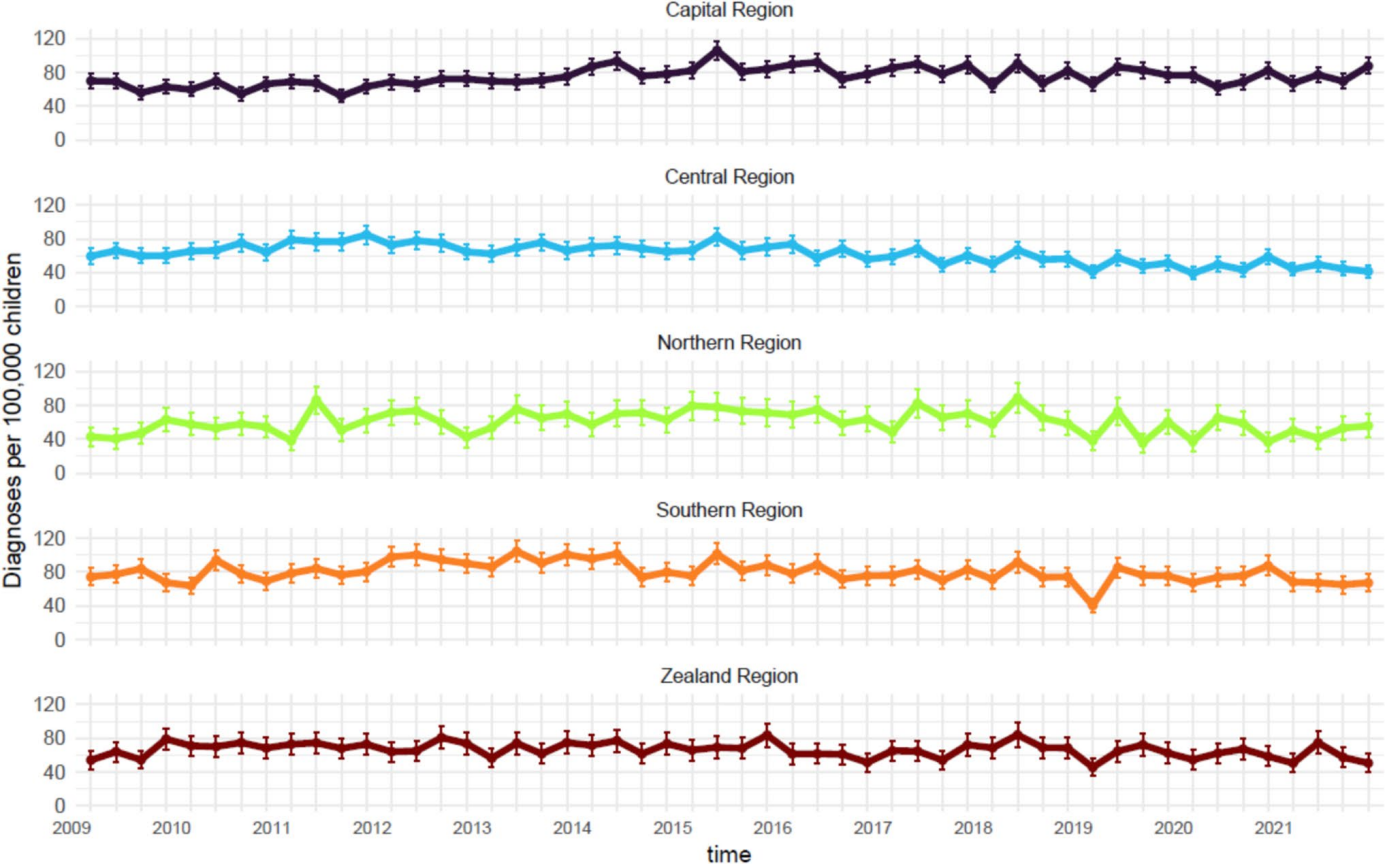
Annual quarterly incidence rates of spine-related diagnoses stratified and facetted by region (2009–2021)

**Fig. 3:**
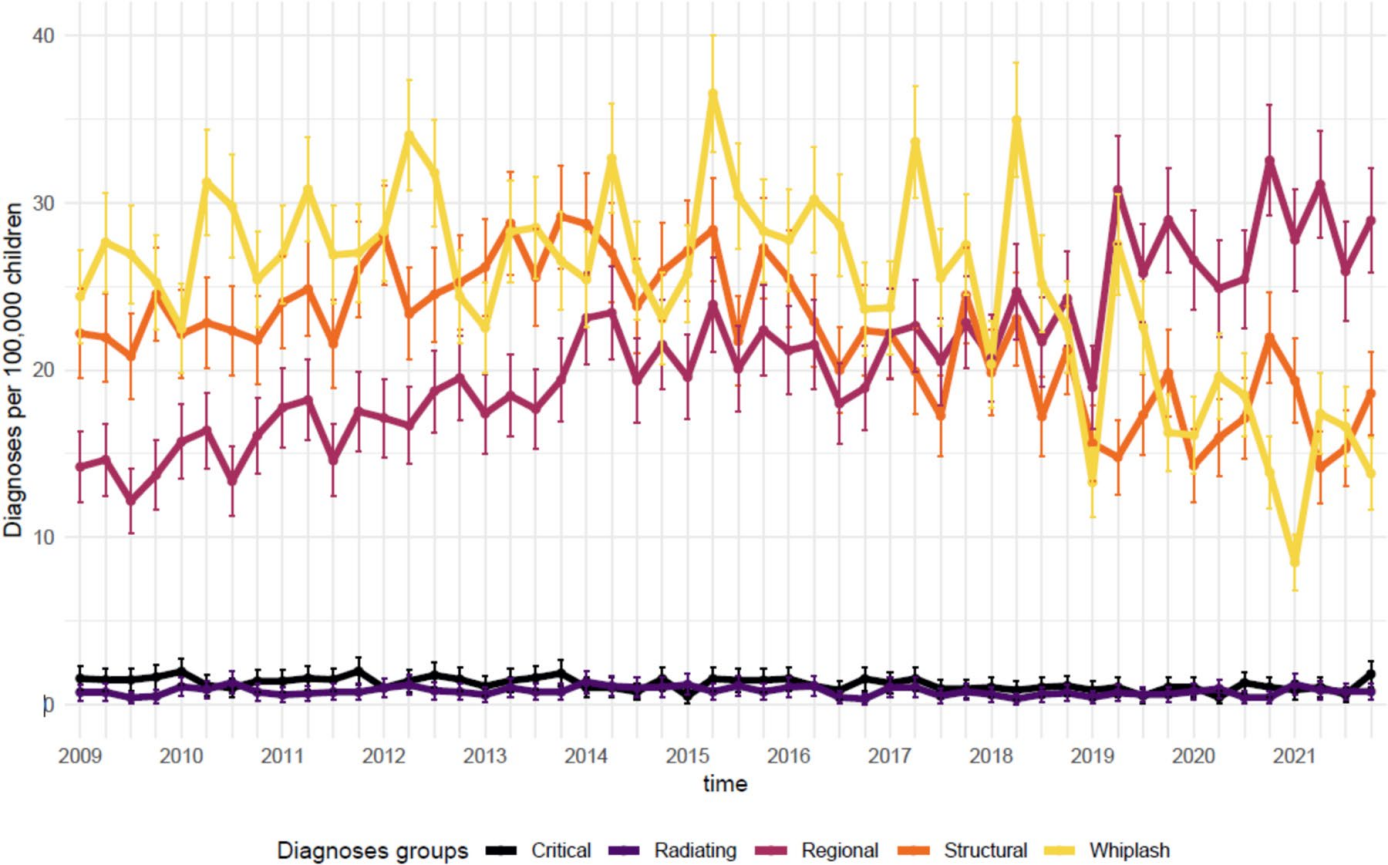
Annual quarterly incidence rates of spine-related diagnoses stratified by diagnostic groups (2009–2021)

**Fig. 4:**
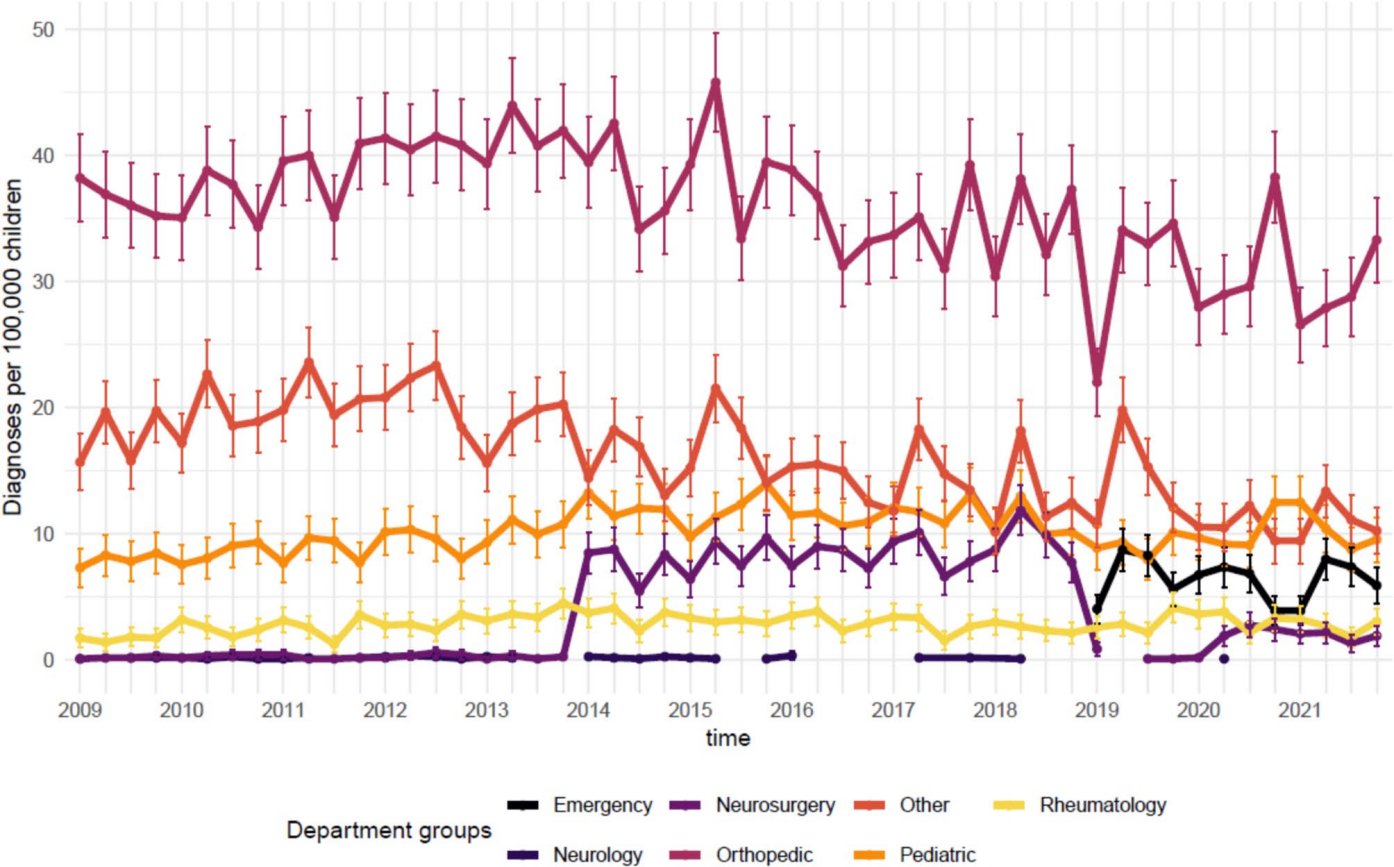
Annual quarterly incidence rates by of spine-related diagnoses stratified by hospital departments (2009–2021)

Overall, the IR ranged from 63 (59 to 68 95%CI) per 100,000 children in 1^st^ quarter of 2009 to 64 (59 to 69 95%CI) per 100,000 children in the 4^th^ quarter of 2021. While variability was observed, the IR generally showed a gradual increase until 2015, followed by a decrease (Fig. 1). Additionally, IRs peaked in the first quarter of each year, except for 2020. Stratification by region showed noteworthy differences in incidence rates, with the Capital and Southern regions reporting nearly twice the incidence compared to Zealand (Fig. 2). However, changes over time were relatively minor across all regions. Stratification was performed by diagnostic groups; we saw greater variation in IR numbers over time. Whiplash and structural diagnoses declined after 2015, whereas regional diagnoses increased steadily over the study period (Fig. 3). Whiplash diagnoses fell from ~30/100,000 in 2018 to ~15/100,000 in 2021, while regional diagnoses stabilized at ~30/100,000 after 2019. Radiating and critical diagnoses are rarely provided and did not change from 2009 to 2021. When stratified by hospital departments, the three most common were Orthopedic, Other, and Pediatric. The Pediatric department had a minor increasing trend, while the Orthopedic and the Other categories tended toward a decrease over time (Fig. 4). A notably fluctuation in diagnoses given by Neurosurgery departments occurred in 2013, and Emergency department diagnoses appeared more frequently from 2019.

## Discussion

This study is the first comprehensive overview of IRs for spine-related diagnoses in Danish children and adolescents over a 13-year period. Children are rarely diagnosed with spine-related conditions in hospital departments, with an approximately average 63/100,000 incident cases per annual quarter, though IRs fluctuated between 50 and 90 per 100,000 children over the study period. When diagnoses are provided, they are often related to whiplash, which was the most common diagnostic group until 2018, after which it declined. This decline aligns with observations from Australia, where a similar decline in whiplash diagnoses was seen in motor vehicle accident insurance data around the same period [22]. Possible explanations include decreased clinical awareness, changes in diagnostic or reporting practices, or shifts in healthcare policies regarding whiplash-related conditions. Likewise, structural diagnoses were very common but also declined after 2015. This pattern may reflect a range of factors, including an actual decrease in such cases, a shift in diagnostic coding preferences, or changes in referral and access to hospital care. Without further data, the exact drivers of this trend remain uncertain. On the contrary, regional diagnoses (e.g., non-specific spinal pain) increased quite substantially during the study period, stabilizing at ~30/100,000 after 2019. The increased use of non-specific diagnostic codes aligns with the broader recognition of the multifactorial nature of spinal pain. This development may reflect a gradual shift toward a biopsychosocial understanding of spinal pain in clinical practice, where biomedical explanations are supplemented by attention to psychological and social dimensions. While our data do not allow us to examine clinical reasoning directly, the coding trend may signal increased acceptance of non-specific pain presentations as legitimate diagnoses. Such a shift is in line with current international guidelines and may influence how children and families experience clinical encounters and expectations of care [23, 24]. Although we are not aware of any specific national policy or guideline changes in Denmark to confirm such a shift in clinical orientation, the recent update of the WHO’s ICD-11 reflects an international movement toward a biopsychosocial approach to pain classification. ICD-11 introduces the category of chronic primary pain, recognizing pain as a disease entity rather than merely a symptom. This framework emphasizes the role of psychological and social dimensions in pain experiences, which may influence clinical documentation practices even in settings where ICD-11 has not yet been adopted.

Additionally, the emergence of emergency department diagnosis from 2019 and onwards highlights potential shifts in healthcare access that should be explored further.

The non-specificity and the impact of social and psychological factors have received much attention in spinal pain over recent years in national and international guidelines [25] and large media coverage in relation to the publication of the *Lancet* Low Back Pain series [26–28]. While these trends primarily concern adults, it is possible that as children grow older, their diagnostic patterns begin to resemble those seen in adults. This could be a potential reason for the apparent shift toward more non-specific codes. However, given the study design, we cannot define it as shifts in clinical practice rather than administrative priorities or other factors.

Future studies should focus on investigating whether the diagnostic coding practices used in Danish hospital departments have any consequences for children. In adults with low back pain, the “label” which is associated with the diagnostic codes does appear to impact people, and sometimes negatively [29]. It would be valuable to investigate how children diagnosed with spine-related conditions move through the healthcare system, from primary to secondary care, and back. Comparing healthcare trajectories between children with and without spine-related diagnoses could provide insights into differences in management and treatment. Still, variability in diagnostic practices may lead to different expectations or outcomes for children, highlighting the broader need for standardizing coding protocols [30]. While the rise in non-specific diagnoses has been linked [30, 31] to referral patterns in adults, its implications for children remain uncertain.

By excluding children with spinal pain diagnoses from 2007 and 2008, we minimized selection bias and aimed for the inclusion of true incident cases. However, some children may still have had earlier spine-related diagnoses. The classification of diagnostic groups was designed to be clinically relevant but may not comprehensively represent all spine-related conditions. Moreover, some B-diagnoses may represent transient symptoms that do not require further examination or follow-up, leading to overrepresentation in our findings.

The choice and specificity of diagnostic coding may influence how clinicians approach treatment planning and referrals, particularly when coding guides reimbursement, triage urgency, or access to specialist services. Non-specific diagnoses, while clinically valid, may also shape family expectations, follow-up patterns, and resource utilization [32]. Clarifying and standardizing coding practices could therefore have important implications for clinical decision-making and equity in care delivery.

The observed regional differences in incidence rates may reflect variations in clinical culture, resource availability, or coding practices across hospital regions. These disparities raise questions about consistency in care and equity in access. Addressing such variation through national coding standards or harmonized clinical pathways may help reduce unwarranted differences in diagnostic activity and ensure more uniform service delivery.

This study was motivated by a lack of knowledge about how spine-related diagnoses are distributed and coded in hospital settings for children. Our findings suggest that diagnostic practices have changed over time and that regional differences persist—even within a small, high-income country with universal healthcare access and coordinated pediatric care pathways. These insights may inform clinical training, resource allocation, and the development of standardized diagnostic frameworks. Given the degree of variation observed in Denmark, similar or greater inconsistencies may exist in other countries with more fragmented health systems. Future research could explore the impact of diagnostic labeling on health outcomes, the patient journey across primary and secondary care, and the potential implications of non-specific coding for treatment decisions and parental perceptions.

## Conclusion

This study is the first comprehensive analysis of the incidence rates for spine-related hospital diagnoses in Danish children. A shift in diagnostic coding practices was indicated, with a decrease in specific structural and whiplash diagnoses and an increase in more “non-specific” diagnoses. Despite changes in coding practices, the overall incidence rates remained stable throughout.

The findings could be interpreted to support a change in the understanding of pediatric spine–related conditions, more in line with a biopsychosocial framework for spinal pain. Future research should focus on understanding what diagnostic coding practices means to children, and whether a diagnosis in hospital departments has any long-term implications.

## Supplementary Information

Below is the link to the electronic supplementary material.

Supplementary file 1 (DOCX 21 KB)

## Data Availability

No datasets were generated or analysed during the current study.

## Appendix 1

Diagnoses codes divided by four groups

**Table:** 184 unique diagnostic codes

Diagnosis code	Diagnostic group
M40.x	Structural
M41.x	Structural
M42.x	Critical
M43.0	Regional
M43.1	Regional
M43.2x	Structural
M43.	Radiating
M43.4x	Critical
M43.5	Critical
M43.6	Structural
M43.8	Structural
M43.9	Structural
M45.	Critical
M46.x	Critical
M47	Regional
M47.0A	Radiating
M47.1x	Radiating
M47.2x	Radiating
M47.8x	Regional
M48	Regional
M48.0	Radiating
M48.1x	Structural
M48.2	Regional
M48.3	Critical
M48.4	Critical
M48.5	Critical
M48.8	Critical
M48.9	Critical
M49.x	Critical
M50.x	Radiating
M51	Regional
M51.0x	Radiating
M51.1x	Radiating
M51.2x	Regional
M51.3x	Regional
M51.4x	Regional
M51.8x	Regional
M51.	Regional
M53	Regional
M53.0x	Regional
M53.	Radiating
M53.3x	Regional
M53.8	Regional
M53.9	Regional
M53.2x	Critical
M54	Regional
M54.0x	Critical
M54.1x	Radiating
M54.2	Regional
M54.3	Radiating
M54.4	Radiating
M54.5	Regional
M54.	Regional
M54.8	Regional
M54.9	Regional
M96	Critical
M96.0x	Critical
M96.1	Critical
M96.2	Critical
M96.5	Critical
M96.6	Critical
M96.8	Critical
M96.9x	Critical
M99.	Regional
M99	Regional
M99.1	Regional
M99.2	Radiating
M99.3	Radiating
M99.5	Radiating
M99.6	Radiating
M99.	Radiating
M99.8	Regional
99.9	Regional
S13	Whiplash
S13.0	Whiplash
S13.1x	Whiplash
S13.2x	Whiplash
S13.3x	Whiplash
S13.4x	Whiplash
S13.5	Whiplash
S13.6	Whiplash

## Appendix 2

Departments:

Orthopedic

- Orthopedic departments

Pediatric

- Pediatric departments
- Child psychiatry departments

Emergency

- Emergency departments

Rheumatology

- Rheumatology departments

Neurology

- Neurology departments
- Neurophysiology departments

Neurosurgery 

- Neurosurgery departments

Other (departments of…)

- Neurosurgery departments
- Cardiology
- Combined
- Dentistry
- Dermato-venerology
- Endocrinology
- General medicine
- Genetic
- Geriatric
- Gynecology obstetric
- Hematology
- Infectious diseases
- Internal medicine
- Medical gastroenterology
- Nephrology
- Nephrology
- Occupational medicine
- Ophthalmology
- Otorhinolaryngology
- Urology
- Physiology nuclear medicine
- Pulmonology
- Physio- and occupational therapy
- Psychiatry
- Radiology
- Surgery
- Surgical gastroenterology
- Surgical gastroenterology
- Plastic surgery
- Vascular surgery

## Abbreviations

CPR: Central Person Register
CRS: Danish Civil Registration System
DNPR: Danish National Patient Register
DST: Statistics Denmark
GP: General practitioner
ICD-10: International Classification of Diseases, 10th Revision
IQR: Interquartile range
IR: Incidence rate
STROBE: Strengthening the Reporting of Observational Studies in Epidemiology
