# Supplementary material for: Incidence of spine-related diagnoses in Danish children: a nationwide registry-based study of hospital data
Source: Eur J Pediatr. 2025 Jun 21;184(7):432. doi: 10.1007/s00431-025-06247-w (PMC12182461; doi:10.1007/s00431-025-06247-w)
Supplement: Supplementary file 1 — (DOCX 21 KB) [file 431_2025_6247_MOESM1_ESM.docx]

Number of diagnoses in the divided specialty

| **Department group** | **Department** | **Diagnoses** |
| --- | --- | --- |
| Orthopedic |  | 22,881 |
| Pediatric |  |  |
|  | Pediatric | 8,546 |
|  | Child psychiatry departments | 1,115 |
| Emergency |  | 905 |
| Rheumatology |  | 2,665 |
| Neurology |  |  |
|  | Neurology departments | 156 |
|  | Neurophysiology departments | 3 |
| Neurosurgery |  |  |
|  | Neurosurgery departments | 2,748 |
| Other |  |  |
|  | Anesthesiology | 65 |
|  | Cardiology | 40 |
|  | Combined | 383 |
|  | Dentistry | 12 |
|  | Dermato-venerology | 12 |
|  | Endocrinology | 20 |
|  | General medicine | 495 |
|  | Genetic | 8 |
|  | Geriatric | 22 |
|  | Gynecology obstetric | 62 |
|  | Hematology | 15 |
|  | Infectious diseases | 58 |
|  | Internal medicine | 269 |
|  | Medical gastroenterology | 26 |
|  | Nephrology | 8 |
|  | No specialty specified | 832 |
|  | Occupational medicine | 73 |
|  | Ophthalmology | 3 |
|  | Otorhinolaryngology | 10 |
|  | Urology | 8 |
|  | Physiology nuclear medicine | 15 |
|  | Pulmonology | 78 |
|  | Physio- and occupational therapy | 271 |
|  | Psychiatry | 104 |
|  | Radiology | 344 |
|  | Surgery | 461 |
|  | Surgical gastroenterology | 35 |
|  | Thoracic surgery | 221 |
|  | Plastic surgery | 49 |
|  | Vascular surgery | 55 |
